# Native adiponectin plays a role in the adipocyte-mediated conversion of fibroblasts to myofibroblasts

**DOI:** 10.1098/rsif.2023.0004

**Published:** 2023-05-03

**Authors:** Mariam Y. El-Hattab, Noah Sinclair, Jesse N. Liszewski, Michael V. Schrodt, Jacob Herrmann, Aloysius J. Klingelhutz, Edward A. Sander, James A. Ankrum

**Affiliations:** ^1^ Roy J. Carver Department of Biomedical Engineering, College of Engineering, University of Iowa, Iowa City, IA 52242, USA; ^2^ Department of Microbiology, Carver College of Medicine, University of Iowa, Iowa City, IA 52242, USA; ^3^ Department of Orthopedics and Rehabilitation, Carver College of Medicine, University of Iowa, Iowa City, IA 52242, USA; ^4^ University of Iowa Fraternal Order of Eagles Diabetes Research Center, Iowa City 52242, IA, USA

**Keywords:** adiponectin, adipocytes, myofibroblasts, α-SMA, fractionation

## Abstract

Adipocytes regulate tissues through production of adipokines that can act both locally and systemically. Adipocytes also have been found to play a critical role in regulating the healing process. To better understand this role, we developed a three-dimensional human adipocyte spheroid system that has an adipokine profile similar to *in vivo* adipose tissues. Previously, we found that conditioned medium from these spheroids induces human dermal fibroblast conversion into highly contractile, collagen-producing myofibroblasts through a transforming growth factor beta-1 (TGF-β1) independent pathway. Here, we sought to identify how mature adipocytes signal to dermal fibroblasts through adipokines to induce myofibroblast conversion. By using molecular weight fractionation, heat inactivation and lipid depletion, we determined mature adipocytes secrete a factor that is 30–100 kDa, heat labile and lipid associated that induces myofibroblast conversion. We also show that the depletion of the adipokine adiponectin, which fits those physico-chemical parameters, eliminates the ability of adipocyte-conditioned media to induce fibroblast to myofibroblast conversion. Interestingly, native adiponectin secreted by cultured adipocytes consistently elicited a stronger level of α-smooth muscle actin expression than exogenously added adiponectin. Thus, adiponectin secreted by mature adipocytes induces fibroblast to myofibroblast conversion and may lead to a phenotype of myofibroblasts distinct from TGF-β1-induced myofibroblasts.

## 1. Introduction

Fibroblasts are viewed as central players in wound healing. After injury, fibroblasts, in response to biochemical factors, such as transforming growth factor beta-1 (TGF-β1), and mechanical factors such as tensile forces in the extracellular matrix (ECM), differentiate into a highly contractile myofibroblast phenotype that rapidly remodels and repairs the tissue [1]. Normally, myofibroblasts disappear at the conclusion of healing. However, chronically activated myofibroblasts persist at the wound site and are associated with excessive collagen production and tissue contracture. Consequently, myofibroblasts remain a common target of therapies designed to prevent scar formation [2].

A growing body of evidence indicates that adipocytes also contribute to tissue repair and restoration both directly and indirectly through interactions with other resident cells in the wound, including fibroblasts [3–5]. Lineage tracing in mice has revealed that adipocytes quickly repopulate the wound site, secrete growth factors and deposit ECM proteins, all of which influences fibroblast activity and healing outcomes [5,6]. In fact, inhibition of adipogenesis was found to significantly decrease fibroblast density and activity in healing wounds, which suggests that interactions between adipocytes and fibroblasts are an essential component of the healing process [5]. However, the mechanisms by which adipocytes influence fibroblast/myofibroblasts during ECM remodelling remain to be elucidated.

In order to identify these mechanisms, we developed a three-dimensional human adipocyte spheroid culture system and investigated the influence of adipocyte-conditioned medium (ACM) on fibroblast behaviour. We found that secreted factors contained within ACM improved wound healing in an *in vitro* scratch assay and converted human dermal fibroblasts to α-smooth muscle actin (α-SMA) positive, contractile, collagen-producing myofibroblasts [7]. Interestingly, ACM-induced myofibroblasts produced significantly more collagen than TGF-β1-induced myofibroblasts [7]. Furthermore, we determined that this conversion, which did not happen in the presence of pre-adipocyte-conditioned medium (PCM), occurred though a TGF-β1-independent mechanism, as small molecule inhibition of the TGF-β1 receptor did not significantly diminish fibrin gel compaction in ACM-treated samples. Since this conversion does not appear to be TGF-β1 dependent, the resultant myofibroblasts are probably distinct in their tissue remodelling potential. However, the identity of the adipocyte-secreted factor responsible for conversion remains unknown.

Here, we report on our progress in identifying the factor responsible for adipocyte-mediated myofibroblast conversion with a specific emphasis on the role that the adipokine adiponectin could be playing. Since the behaviour of myofibroblasts are determined in part by the factors that induce their differentiation, uncovering the mechanisms by which adipocyte-secreted factors induce myofibroblast conversion is a critical first step to determining if adipocytes generate myofibroblasts that are scar forming versus tissue regenerating.

## 2. Results

### 2.1. Conditioned medium from primary adipose-derived cells induces myofibroblast conversion

**Figure 1.**
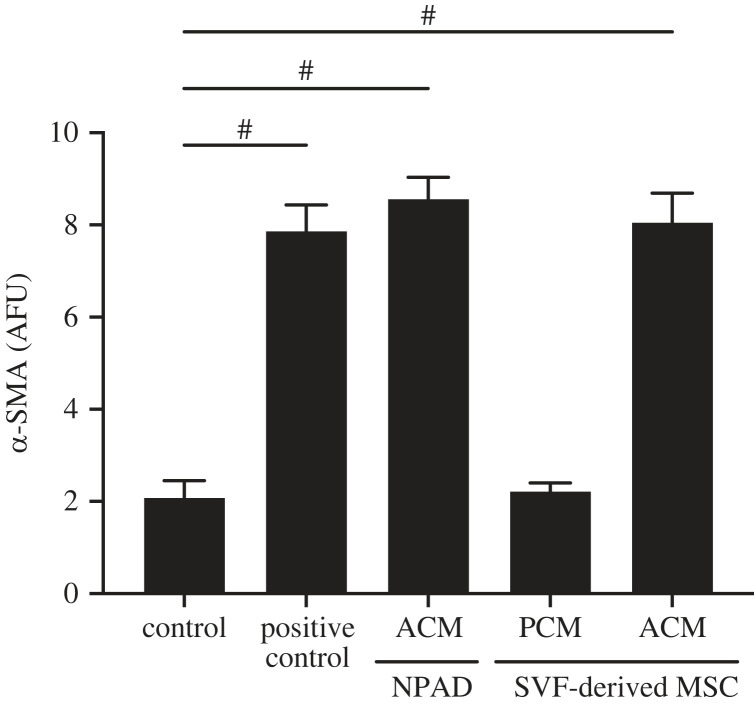
ACM from SVF-derived cultures induces myofibroblast conversion similarly to immortalized NPAD-derived cultures. ACM and PCM from SVF-derived MSC cultures were assessed for their ability to induce human fibroblast expression of α-SMA. ACM collected from immortalized NPAD-derived cultures and positive control media containing TGF-β1 and ascorbic acid were used as controls. α-SMA expression was measured using a fluorescent plate reader after immunolabelling. One-way ANOVA with Dunnett's multiple comparison relative to control ^#^*p* < 0.0001, Data are mean ± s.d., *N* = 3.

We reported previously on the myofibroblast-inducing potential of ACM derived from an immortalized human pre-adipocyte cell line (NPAD) [7]. To determine if this potential is also present in spheroids formed from primary human cells, we collected conditioned media from primary cells and quantified myofibroblast conversion similar to our previous studies [7–9]. Adipose mesenchymal stromal cells isolated from the stromal vascular fraction (SVF) of digested donor adipose tissue were differentiated in spheroid cultures into either pre-adipocytes or adipocytes. After 10 days of differentiation, conditioned media was collected and assessed for its ability to induce myofibroblast conversion. We found ACM derived from both the immortalized NPAD-derived and the primary SVF-derived cultures significantly induced α-SMA levels above the control and similar to the positive control (figure 1). Consistent with our prior studies, PCM did not increase α-SMA expression (figure 1). Thus, primary SVF-derived human adipocytes also induce myofibroblast conversion, a finding that gives us confidence that this signalling mechanism is a general feature of our three-dimensional spheroid culture system.

### 2.2. Adipocyte- and pre-adipocyte-conditioned media differ substantially in adipokine profile

**Figure 2.**
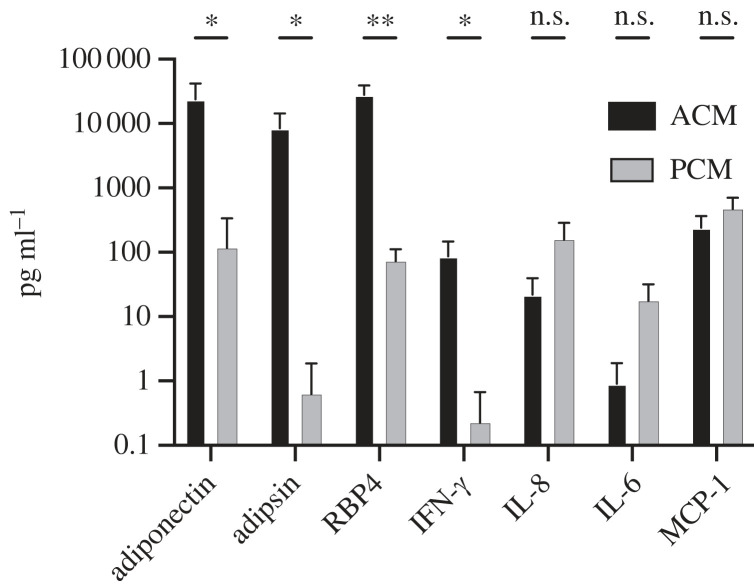
The adipokine profile between ACM and PCM differs significantly. Adipokine profile of ACM and PCM as measured using a bead-based adipokine array. Each cytokine was compared using unpaired *t*-tests, two-tailed, *p < 0.05, **p < 0.005. Data are mean ± s.d., *N* = 4.

Having established that ACM from primary cultures induces myofibroblast conversion while PCM does not, we investigated what factor(s) are present in ACM but not PCM that could potentially be responsible. We began by comparing the adipokine content of human SVF-derived ACM and PCM. Using a LEGENDplex™ Human Adipokine Panel (13-plex), we analysed ACM and PCM for adiponectin, adipsin, retinol-binding protein-4 (RBP4), monocyte chemoattractant protein 1 (MCP-1), interleukin 1 beta (IL-1*β*), interferon gamma-induced protein 10 (IP-10), interleukin 10 (IL-10), interleukin 8 (IL-8), leptin, interleukin 6 (IL-6), interferon gamma (IFN-γ), resistin and tumour necrosis factor alpha (TNF-α). Of the 13 factors measured, six were found to be at or below the detection limit (DL) of the assay for all samples: IL-1*β* (DL: 0.24 pg ml^−1^), IP-10 (DL: 14.5 pg ml^−1^), IL-10 (DL: 0.28 pg ml^−1^), leptin (DL: 1 pg ml^−1^), resistin (DL: 1.2 pg ml^−1^) and TNF-α (DL: 0.5 pg ml^−1^). Of the seven adipokines above the DL, we identified four adipokines that differed significantly and by orders of magnitude between ACM and PCM (figure 2). ACM had levels of adiponectin, adipsin, RBP4 and IFN-γ that were 10–100 times higher than PCM. By contrast, PCM had slightly higher levels of IL-8 and IL-6, but the experiment was not powered enough to declare the differences in IL-8 and IL-6 statistically significant (*p* = 0.078 and *p* = 0.054, respectively). Thus, elevated adiponectin, adipsin, RBP4 and IFN-γ are all candidate adipokines that could be playing a role in ACM-mediated induction of myofibroblast conversion.

## 3. Fractionation of adipocyte-conditioned medium reveals factor physico-chemical properties consistent with adiponectin

**Figure 3.**
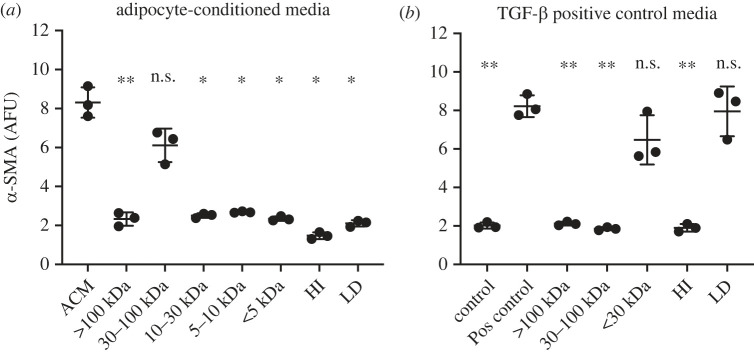
Fractionation of ACM reveals the inducing factor is between 30 and 100 kDa, heat labile and lipid associated. (*a*) ACM fractionation shows a molecule(s) in the 30–100 kDa fraction involved in fibroblast conversion to myofibroblasts. Heat inactivation (HI) and lipid depletion (LD) both removed this effect. (*b*) TGF-β1 positive control fractionation shows that the TGF-β1 was retained in the less than 30 kDa and LD fractions. Myofibroblast conversion measured via fluorescently labelled α-SMA. Data are mean ± s.d., *N* = 3. One-way ANOVA conducted with Dunnett's multiple comparisons test. *p < 0.05, **p < 0.005; AFU: arbitrary fluorescence units.

Having found substantial differences between the adipokine profiles of PCM and ACM, we next performed a physico-chemical characterization of conditioned media to help narrow candidate factors responsible for myofibroblast conversion. Since the adipokines elevated in ACM have different molecular weights (adiponectin – monomeric 30 kDa, trimeric 90kda; adipsin—28 kDa; RBP4—21 kDa; IFN-γ—17 kDa, IL-6—21 kDa; IL-8—8.4 kDa), we fractionated the ACM using molecular weight cut-off (MWCO) filters in the ranges of less than 5 kDa, 5–10 kDa, 10–30 kDa, 30–100 kDa and greater than 100 kDa and then repeated the α-SMA assay. We found that after fractionation, the ability of ACM to induce α-SMA expression was lost in all fractions except the 30–100 kDa fraction (figure 3*a*). Thus, we conclude that the factor could be adiponectin, but that it is not adipsin, RBP4, IFN-γ, IL-6 or IL-8.

To determine if the factor is a protein, we heat inactivated (HI) the ACM and found that doing so completely eliminated the ability of ACM to induce α-SMA expression (figure 3*a*). Finally, to determine if the factor is lipid associated, we depleted lipids (LD) and lipid-associated factors using Cleanascite and found the α-SMA inducing activity of ACM was also eliminated (figure 3*a*). When fractionation, heat inactivation or lipid depletion was performed on TGF-β1 containing positive control media, only the less than 30 kDa fraction and lipid-depleted ACM retained α-SMA inducing activity, findings completely expected and consistent with the physico-chemical properties of TGF-β1 (the active form of TGF-β1 is a 25 kDa dimer). Thus, the factor in ACM responsible for inducing myofibroblast conversion shows distinct physico-chemical characteristics compared with TGB-β1 containing positive control media, which is an additional confirmation of our earlier study [7].

The observation that both lipid depletion and heat inactivation eliminated myofibroblast conversion suggests that both a lipid and a heat-labile protein could be involved. To test for this possibility, we combined ACM HI, which should retain the lipids, and ACM LD, which should retain the proteins, and tested for myofibroblast conversion. We again found ACM HI and ACM LD alone eliminated myofibroblast conversion but combining ACM HI and ACM LD also did not restore any measurable increase in α-SMA induction (electronic supplementary material, figure S1). While this experiment does not eliminate the possibility that multiple factors are involved, it does raise the possibility that the protein itself could also be lipid associated, as it appears to be removed/deactivated during lipid depletion.

### 3.1. High concentrations of exogenously added adiponectin can induce myofibroblast conversion

**Figure 4.**
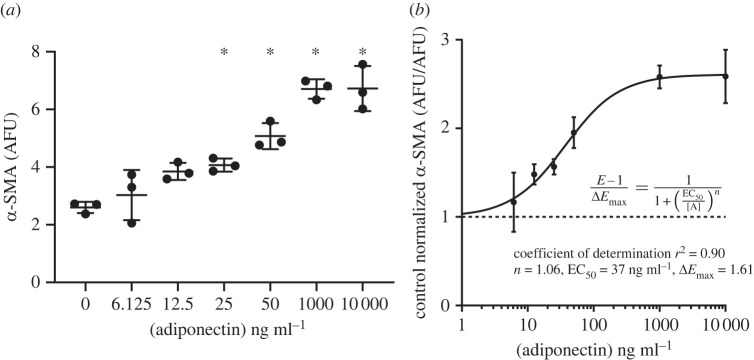
Human adiponectin promotes the expression of α-SMA from fibroblasts in a dose-dependent manner. (*a*) Myofibroblast conversion measured via fluorescent immunolabelling α-SMA in fibroblasts 48 h after treatment with increasing concentrations of human adiponectin. (*b*) To fit a dose–response curve, control-normalized α-SMA expression (*E*) in response to each adiponectin concentration ([A]) was computed as the ratio of labelled α-SMA fluorescence to the mean response in the control group with [A] = 0. The change in α-SMA expression above the mean control response (*E*–1) was fit by a Hill equation using a least-squares regression, yielding three parameter estimates: the maximal change in expression (Δ*E*_max_), the Hill coefficient (*n*) and the half-maximal effective concentration (EC_50_). Data are mean ± s.d., *N* = 3. One-way ANOVA conducted with Dunnett compared with 0 adiponectin control, *p < 0.05.

Of the four substantially elevated adipokines we identified in ACM compared with PCM, only adiponectin conforms to the 30–100 kDa range where myofibroblast conversion was observed. Adiponectin is a complex molecule with a monomeric molecular weight of approximately 30 kDa. It has three secreted oligomeric forms: a low molecular weight (LMW) trimer that is 67 kDa, a middle molecular weight (MMW) hexamer that is 140 kDa and a high molecular weight (HMW) multimer that is at least 300 kDa monomers [10,11]. A bioactive, proteolytically degraded globular form that consists of the trimeric head domain minus the stalk also exists natively [12]. To add further complexity, adiponectin also can bind anionic phospholipids [13]. Based on the physico-chemical properties of the unknown factor in ACM, we sought to determine if adiponectin could be the factor in ACM responsible for myofibroblast conversion by performing a dose–response experiment using exogenously added human adiponectin (ex-Adip). We found ex-Adip at concentrations of 25 ng ml^−1^ and above induced significant myofibroblast conversion in a dose-dependent manner (figure 4*a*). Fitting a dose–response curve to the data revealed a half-maximal effective concentration (EC_50_) of 37 ng ml^−1^ [14]. Although these data indicate that ex-Adip can induce myofibroblast conversion, the calculated EC_50_ value for ex-Adip is 2–4 times higher than the concentration of native adiponectin measured in ACM.

### 3.2. Adiponectin at concentration found in adipocyte-conditioned medium only weakly induces myofibroblast conversion

**Figure 5.**
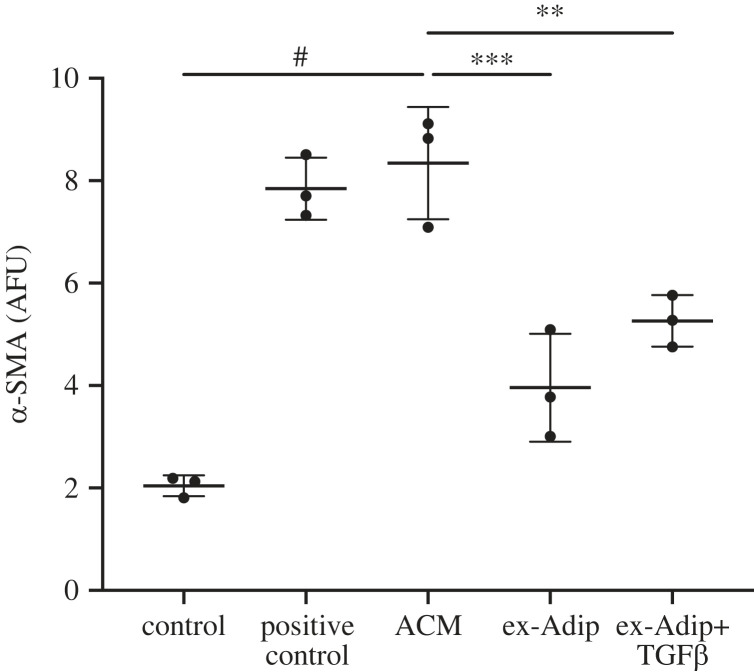
Exogenous adiponectin and TGF-β1 does not account for ACM-mediated induction of myofibroblasts. Human adiponectin alone (ex-Adip) or adiponectin with TGF-β1 (ex-Adip + TGF*β*) were added to 0.5% FBS DMEM in the same concentrations measured in ACM (approx. 10 ng ml^−1^ and 270 pg ml^−1^, respectively) to determine if they synergistically contribute to myofibroblast conversion. Data are mean ± s.d., *N* = 3. One-way ANOVA with Dunnett's multiple comparison compared with ACM, ^#^*p* < 0.0001, ***p < 0.001 **p < 0.01.

We sought to determine if we could replicate the same level of α-SMA expression using ex-Adip at the same concentration native adiponectin is found in ACM (approx. 10 ng ml^−1^). While ACM again induced significant and robust α-SMA expression, ex-Adip induced significantly less myofibroblast conversion (figure 5). Because ACM also contains approximately 270 pg ml^−1^ of TGF-β1, we also tested whether there is a synergy between adiponectin and TGF-β1 by matching both adiponectin and TGF-β1 concentrations in ACM using ex-Adip and recombinant TGF-β1. This combination also failed to replicate the same level of myofibroblast conversion observed with ACM (figure 5). Thus, while ex-Adip is able to induce myofibroblast conversion, it fails to account for the full myofibroblast-inducing potential of ACM. One potential explanation is that ex-Adip differs in form and/or lipid binding partners compared with the adiponectin in ACM. SDS-PAGE from the supplier shows several distinct bands, which suggests that ex-Adip contains a mix of different adiponectin forms and/or post-translational modifications.

Alternatively, ACM may include additional factors that work in concert with adiponectin to induce myofibroblast conversion. Other commonly studied adipokines [15] not included in the 13-plex adipokine assay, such as fibroblast growth factor 21 (FGF21), and dipeptidyl peptidase 4 (DPP-4) do not fit the molecular weight profile of the unknown factor. BMP-4 (46 kDa) and BMP-7 (49 kDa) fit the molecular weight profile, but are, respectively, either secreted by both pre-adipocytes and adipocytes [16] or have functions associated with energy expenditure and adipocyte browning [15], and thus are unlikely candidates.

Mass spectrometry-based proteomic studies of adipocyte cell and tissue secretomes have identified hundreds of differentially expressed proteins (see Gianazza *et al*. [17] for a recent comprehensive review on the subject). The most relevant study for our work is on proteomic profiling of conditioned medium collected from primary human adipocytes [18]. In that study, 347 proteins were identified, 263 of which were predicted to be secreted. Of those proteins, 139 had molecular weights ranging between 30 kDa and 100 kDa. Performing a similar type of proteomic profiling of both ACM and PCM should enable us to identify proteins that are present in ACM and not in PCM that could be contributing to the myofibroblast conversion observed here.

### 3.3. Depletion of native adiponectin from adipocyte-conditioned medium significantly reduces myofibroblast conversion

**Figure 6.**
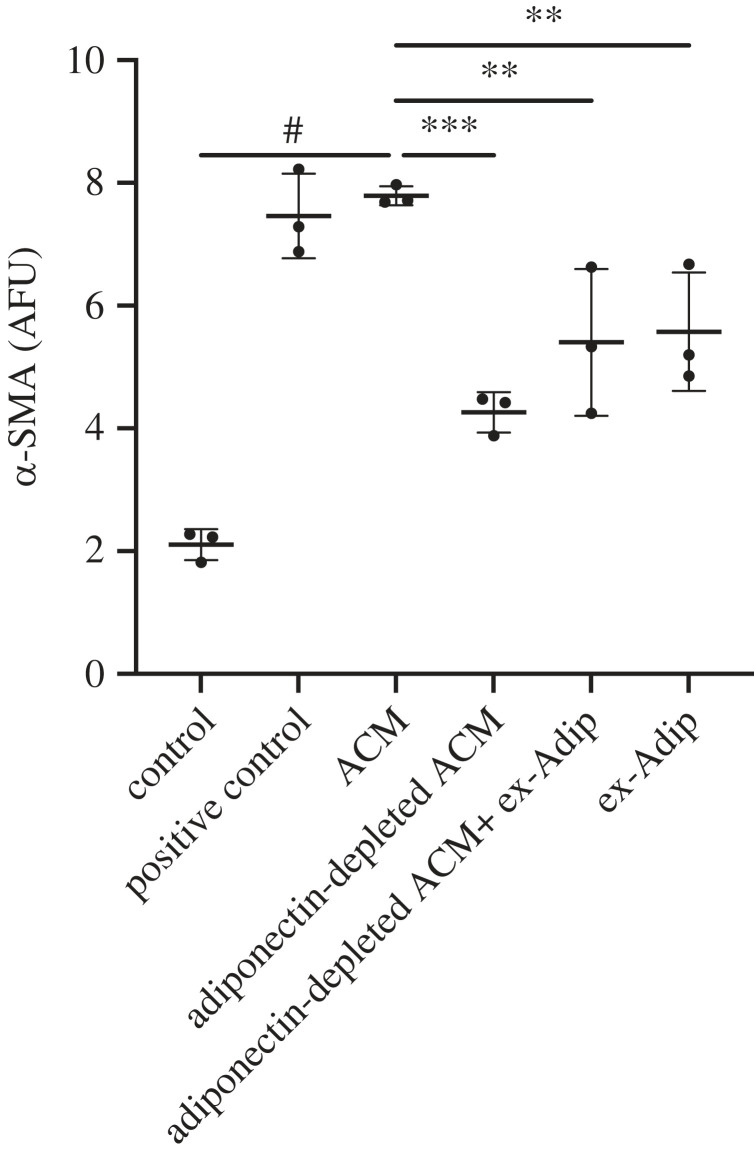
The depletion of native adiponectin from ACM substantially reduces ACM's ability to induce myofibroblast conversion. ACM was depleted of adiponectin (adiponectin-depleted ACM) using magnetic beads coated with anti-adiponectin antibodies and then applied to fibroblasts. Human adiponectin (ex-Adip) was added in the same concentrations measured in ACM (approx. 10 ng ml^−1^) to determine if the function of native adiponectin could be replaced with exogenous adiponectin. Data are mean ± s.d., *N* = 3. One-way ANOVA with Dunnett's multiple comparison compared with ACM, ^#^*p* < 0.0001, ***p < 0.001 **p < 0.01.

Since ex-Adip could be different from the adiponectin in ACM, we next asked if the depletion of native adiponectin from ACM would result in a loss of the myofibroblast converting potential. Using anti-adiponectin antibody-coated beads, we depleted adiponectin from our ACM and repeated the α-SMA assay. We found that the depletion of native adiponectin from the ACM resulted in a substantial and significant decline in fibroblast α-SMA expression (figure 6), which suggests that native adiponectin is necessary for ACM to efficiently induce myofibroblast conversion. To test if a second factor in ACM synergizes with adiponectin, we also spiked ex-Adip into the adiponectin-depleted ACM but it was unable to restore α-SMA expression back to levels seen with ACM. This finding suggests that the attributes of native adiponectin (e.g. its form or binding partner) are vital for mediating the conversion of fibroblasts to myofibroblasts. The different forms of adiponectin (e.g. globular, trimers, hexamers and multimers) are thought to each have distinct functions, including as carriers for other molecules, such as lipids [11]. Furthermore, in addition to the total amounts of adiponectin, the distribution of the forms of adiponectin also has been attributed to different downstream biological functions [19].

## 4. Discussion

Adiponectin is most commonly thought of as a hormone that regulates insulin resistance, but it is also associated with anti-inflammatory properties, as plasma levels are inversely correlated with many obesity-related inflammatory diseases [20]. In terms of direct anti-inflammatory effects on fibroblasts, adiponectin (and adiponectin agonists) has been shown to inhibit myofibroblast conversion [21,22], mitigate collagen gene expression in the presence of TGF-β2 [23] and reduce ECM protein synthesis [24], most likely through inhibition of the NF-κB pathway through AMP activated protein kinase (AMPK) signalling [20].

By contrast, several clinical studies have found high adiponectin levels in pro-inflammatory conditions such as rheumatoid arthritis and joint disease [25–27]. Some *in vitro* studies also support a link between adiponectin and cell activities associated with inflammation. Adiponectin can induce α-SMA expression in monocytes [28] and increase collagen production and hyaluronic acid (HA) synthesis in neonatal dermal fibroblasts [29]. There is also evidence that the form of adiponectin can impact fibroblast activity. Akazawa *et al.* found that HA synthesis in human dermal fibroblasts increased in a dose-dependent manner with exposure to recombinant oligomeric adiponectin but not to globular adiponectin [30]. Conflicting evidence on the effects of adiponectin on fibroblast/myofibroblast conversion might be due to a lack of exact details on the source and form of adiponectin used. For example, recombinant adiponectin can be made in either bacterial or mammalian expression systems, which can impact post-translational modifications known to impact adiponectin's bioactivity [31].

The apparent contradictions in adiponectin's activity could also be due to the complexities of the fibroblasts themselves, as (i) fibroblasts have behaviours specific to their tissue of origin [32], (ii) myofibroblasts within a tissue can originate from multiple cell sources [33], and (iii) myofibroblasts within a healing wound can be subdivided into several transcriptionally distinct subpopulations [3,4,34]. In the light of these points, a more nuanced view of myofibroblasts is beginning to emerge. For example, myofibroblasts that originate from dermal adipocytes are enriched for genes associated with various ECM proteins, but not for genes associated with collagen maturation and cross-linking [3]. These results suggest that healing outcomes could be dependent on which myofibroblast subpopulations are present in the healing wound. Therefore, an understanding of how those different subpopulations emerge and what their specific roles are could be beneficial for improving healing and tissue regeneration.

It is also worth noting that plastic and reconstructive surgeons have reported that fat grafting, which contains adipocyte stem cells, pre-adipocytes and adipocytes, can reduce existent scar tissue through mechanisms that remain unknown [35,36]. One possible mode of action is that these cells secrete factors that select for myofibroblast subpopulations that are more conducive to scar resolution. If this proposition holds true, it will be interesting to know if the adipocyte-mediated myofibroblast conversion we have observed, which occurs through a TGF-β1 independent mechanism, leads to a myofibroblast subpopulation that is either pro-scar or pro-regeneration. Such information will be important for optimizing current fat grafting techniques and for developing new therapeutic strategies.

In conclusion, physico-chemical characterization of ACM revealed that the factor responsible for myofibroblast conversion is retained in the 30–100 kDa fraction and loses activity with heat treatment and lipid depletion. Significant differences in the secretion profile of common adipokines were found between ACM and PCM, but of the adipokines we measured, only adiponectin has physico-chemical properties consistent with our findings. For this reason, we investigated whether adding adiponectin at levels comparable to those measured in ACM could induce myofibroblast conversion. We found that fibroblasts are responsive to adiponectin in a dose-dependent manner but at much higher concentrations than those measured in ACM. We also found that depleting native adiponectin from ACM removes its ability to induce myofibroblast conversion, suggesting adiponectin in its native form is important for its activity on fibroblasts. The profile of adipokines produced by adipocytes is diverse and complex, and it is likely that adipocytes secrete multiple factors that modify fibroblast behaviour. However, the data presented here implicate adiponectin as a necessary but not sufficient factor in inducing the unique myofibroblast phenotype we have uncovered.

## 5. Materials and methods

### 5.1. Pre-adipocyte and adipocyte spheroid culture, maintenance and conditioned medium collection

Pre-adipocyte spheroids were generated using a standard hanging drop cell culture as previously described [8]. Spheroids (each containing 20 000 cells) were then transferred to ultra-low adherent 24-well plates (Corning CLS3473), with five spheroids per well. Pre-adipocyte spheroids were maintained in pre-adipocyte growth medium (PGM-2, Lonza) containing 10% fetal bovine serum (FBS), 0.01% gentamycin sulfate/amphotericin B and 200 µM L-glutamine for 10 days. Adipocyte spheroids were formed by culturing spheroids in pre-adipocyte differentiation medium (PDM-2, Lonza) containing 1% insulin, 0.1% 3-isobutyl-1-methylxanthine (IBMX), 0.1% dexamethasone and 0.2% indomethacin for 10 days. After 10 days, medium was removed, the spheroids were rinsed gently with 1X PBS, then 0.75 ml of Dulbecco's modified eagle medium (DMEM, Gibco) containing 0.5% FBS (Gibco 26140079), 1% penicillin/streptomycin (PS, Gibco 15140-122) and 0.1% amphotericin B (AB, Gibco 15290-018) was added to each well. After 2 days, this medium, termed either PCM or ACM, respectively, was collected and stored at −80°C.

### 5.2. Legendplex adipokine panel

PCM and ACM were screened with a multiplex adipokine assay in order to measure levels of common cytokines, hormones and other factors commonly secreted by adipose tissues. Collected medium was processed and run in triplicate via flow cytometry as described by the manufacturer (Biolegend LegendPlex Human Adipokine Panel, 741062).

### 5.3. Protein fractionation of conditioned medium

To fractionate ACM into pools of medium with different MWCO, a 100 kDa MWCO Vivaspin PES filter (Sartorius) was pre-rinsed with deionized water to ensure full saturation of the filter membrane. After the removal of the water, ACM was added and centrifuged in a swing-bucket centrifuge for 8 min at 4000*g* per the recommendation by Sartorius. The column was inverted, re-centrifuged at 3000*g* for 2 min, and the concentrate was resuspended in 0.5% FBS DMEM to the original volume. To fractionate proteins in the 30–100 kDa molecular weight range, an Amicon 30 kDa MWCO (Milipore Sigma) filter was pre-rinsed as described before. The previously collected filtrate was added to the column and centrifuged at 1500*g* for 30 min. The filtrate was collected for additional fractionation steps. The concentrate was again resuspended in 0.5% FBS DMEM to the original volume. This process was repeated twice more with 10 kDa and 5 kDa MWCO Vivaspin PES filters (Sartorius), for a total of five fractions of ACM.

### 5.4. Lipid depletion and heat inactivation

To heat inactivate proteins in the medium, ACM was heated for 60 min at 85°C with a benchtop hotplate. For lipid depletion, ACM samples were treated with Cleanascite HC to remove lipid as described by Castro *et al*. [37]. A 0.5 ml of Cleanascite HC was added to Eppendorf tubes and centrifuged at 1000*g* for 20 min to pellet the beads. The supernatant was removed, and the pellet was resuspended in ACM using a benchtop vortex. Samples were then incubated at 4°C overnight on a rocker plate. The next day, samples were centrifuged at 1000*g* for 45 min to pellet the lipid-bound beads. The lipid-depleted ACM was decanted into a separate Eppendorf tube and filtered through a 0.45 µm pore size filter to remove residual polymer particles.

### 5.5. Quantification of α-smooth muscle actin expression

A 28-year-old human dermal fibroblasts from breast surgical discard (University of Iowa Tissue Procurement Core) between passages 3–5 were plated at 100 000 cells cm^−2^ in black-walled 96-well plates containing 150 µl of DMEM with 10% FBS, 1% penicillin/streptomycin and 0.1% amphotericin B. Fibroblasts were left to attach for 6–8 h before removing the medium, rinsing with 1 x PBS, and replacing with the various treatment groups in triplicate. The treatment groups included ACM and positive control (0.5% FBS in DMEM plus 1 ng ml^−1^ TGF-β1 and 50 µg ml^−1^ ascorbic acid) subjected to molecular weight fractionation in the ranges of greater than 100 kDa, 30–100 kDa, 10–30 kDa, 5–10 kDa and less than 5 kDa, and lipid depleted and HI. Fibroblasts were maintained in a humidified 95%/5% air/CO_2_ incubator at 37°C for 48 h. After treatment, samples were rinsed three times with 1 x PBS, fixed and permeabilized with 4% paraformaldehyde and with 0.1% TritonX-100, respectively. Each well was blocked with 5% BSA-tween solution and incubated with a primary mouse anti-human α-SMA antibody (ab7817, Abcam, Cambridge, UK) solution at a 1 : 100 dilution overnight at 4°C. Samples were blocked again and incubated with two–three drops of an anti-mouse poly-HRP-conjugated secondary antibody from the Invitrogen Tyramide SuperBoost secondary staining kit (ThermoFisher, B40912). Samples were rinsed three times with 1 x PBS, and the tyramide working solution conjugated to AlexaFluor 488 was added and allowed to react for 20 min. Stop solution was then added followed by another three times rinse with 1 x PBS. Cell nuclei were stained using Hoescht 33342 (Invitrogen, H3570) prior to imaging. The percentage of α-SMA positive cells was quantified using on a VarioSkan Lux plate reader. Measurements were made at an excitation/emission of 496/524 nm with each well receiving 29 measurements, each for 1000 ms.

### 5.6. Addition of exogeneous human adiponectin

To investigate the effects of exogeneous adiponectin on dermal fibroblasts, we reconstituted adiponectin from pooled human serum (BioVendor RD16202350) in sterile deionized water to a stock concentration of 0.1 mg ml^−1^. Adiponectin was then added to low serum medium to produce final concentrations ranging from 6.125 ng ml^−1^ to 10 µg ml^−1^ for α-SMA quantification.

### 5.7. Adiponectin depletion from adipocyte-conditioned medium

Using a DynaBeads co-immunoprecipitation kit (Invitrogen 14321D), 2 mg of magnetic beads were vortexed with C1 buffer for 30 s. The beads were collected with a magnet and conjugated with 12 µg of adiponectin antibody (ThermoFisher 19F1) in accord with the Invitrogen protocol. Conjugation was followed by a series of wash and collection steps using the supplied buffers. The final 10 mg ml^−1^ suspension of antibody-coupled beads was washed with 0.1% BSA in 1 x PBS and then resuspended in 3 ml ACM. The solution was incubated at 4°C with constant agitation for 10–30 min. The beads were collected with a magnet and the supernatant (adiponectin-depleted ACM) was removed into a separate tube. Adiponectin depletion was confirmed using an ELISA (less than 0.3 ng ml^−1^, Biolegend Adiponectin ELISA Max, 442304) and then the adiponectin-depleted ACM was used in the α-SMA fluorescence assay to treat fibroblasts. Adiponectin from human serum was also added back to a portion of the adiponectin-depleted ACM (10 ng ml^−1^).

### 5.8. Statistics

All statistical analysis was conducted in GraphPad Prism version 9 (GraphPad, San Diego, CA). Replicates from independent experiments were used to determine individual experiment means, which were then analysed for significance (*p* < 0.05) using either one-way or two-way analysis of variance (ANOVA) followed by either Dunnett's multi-comparison tests or Tukey *post hoc* tests.

## Data Availability

The data used to support the findings of this study are presented in the figures and available from the corresponding author upon request. All data are included within the figures of the paper and raw forms are available upon request to the corresponding author. The data are provided in the electronic supplementary material [38].
